# Lunatics in Unlicensed Houses

**Published:** 1897-05-08

**Authors:** 


### Editor's Letter-Box.

[Our Correspondents are reminded that prolixity is a great bar to publication, and that brevity of style and conciseness of statement greatly facilitate early insertion.]

### LUNATICS IN UNLICENSED HOUSES.

Dr. W. Simpson Craig writes: In the number of The Hospital for April 10th you call attention to the grave dangers to which the public are exposed from the too early discharge of lunatics on "recovery" from asylums. These are great and real, especially in view of their influence upon the race. There is, however, another danger to which the title of your article, "The Making of Madmen," would more aptly apply, as it is in very truth a method by which chronic lunatics are made. I refer to the large and increasing number of the insane, chiefly amongst the upper classes, who are now placed for care and treatment in the charge of ignorant and, as in a case recently reported in The Hospital, unscrupulous persons. The late Lord Shaftesbury foresaw this danger as one of the probable results of increasing the statutory forms required to be signed before placing a private patient in a licensed house, asylum, or hospital. The evil has, however, assumed larger proportions and increased more rapidly than could have been foreseen.

Private patients of both sexes are now received in all sorts of houses by all sorts of people; no consideration is given as to the suitability of the house, its arrangements, or to the qualifications and previous experience of those undertaking the charge. The main factor in the case is the increase of the family's income derived from the payments of the patient. Cases of acute melancholia and violent mania are thus placed. In the one case the patient is too often left to dwell upon the unhappy thoughts which fill the mind, without occupation, amusement, or interest. In the other, the violent excitement demands some controlling measures, and, from ignorance, gross forms of restraint and seclusion are employed. As one having such a charge said the other day, "What could we do but tie her down when she attacks her nurse, breaks the furniture, and tries to get through the window?" So "tied down" she was for hours together. And this is no solitary case. Who is to blame for this state of things at the close of the nineteenth century, when so much has been done for the insane by enlightened legislation and by an important Government Department specially engaged in the supervision of asylums, hospitals, and licensed houses? In many cases relatives are undoubtedly responsible; they prefer a private house rather than the imaginary publicity of even a private asylum, and regard it as more consistent with family feeling to use every means to avoid the stigma which is yet supposed to attach to such as have been legally placed in a "Home" for the care and treatment of the insane. That my professional brethren frequently support this view of the relatives I am bound to admit, though from their better knowledge they should be aware of the valuable time thus lost in the early treatment of the patient, and they cannot be ignorant that recovery is retarded thereby, and the prospect of ultimate cure endangered. The serious abuses which were said to exist in private asylums previous to the appointment of the present Lunacy Commission are very much increased under this irregular method of private care. Under this system there is no supervision, and it is only when a case of flagrant cruelty and neglect is reported in the newspapers that the public indignation is aroused; but there are many sufferers shut up in mute misery with none to help, and these in their helplessness and hopelessness sink into chronic lunatics, "made madmen."

